# Expertly used unsupervised clustering provides clinical tools as well as insight

**DOI:** 10.1093/ehjdh/ztaf015

**Published:** 2025-03-05

**Authors:** Johan De Bie

**Affiliations:** Department of Electrical, Electronic, and Information Engineering ‘Guglielmo Marconi’, University of Bologna, Via Cesare Pavese 50, 47521 Cesena, Italy

## Introduction

In the present issue of the European Heart Journal—Digital Health, Madrid *et al*.^1^ meticulously report on a project in which they used so-called unsupervised machine learning (ML), aka multidimensional clustering, of single-channel ECG features (lead I), to identify distinct groups in a patient cohort with coronary artery disease (CAD). The scope of their work was to investigate the association of these clusters with incident and prevalent heart failure (HF), atrial fibrillation (AF), or ventricular arrhythmias (VA). The authors used two distinct, prospectively collected, and publicly available databases from the UK Biobank^2^ to develop their method and test the results.

The results show two fairly well-separated clusters, and the authors demonstrated a robust association with both incident and prevalent HF in particular.

## Discussion

In recent years, a wealth of artificial intelligence or ML articles has been published related to ECG and heart disease, some in attempts to still find something new and useful in the age-old ECG, some to provide tools to compensate for a decrease in the availability of human expert skill, and maybe some also just because it is possible.^3–5^

The work of Madrid *et al*. provides food for thought and stands out in several aspects.

Firstly, the team clearly has a good knowledge of the ECG, the signal processing involved, as well as scientific and statistical methodology. As much as we admire the results that can be obtained nowadays with sophisticated ML techniques and the progress that has been made in recent years, domain knowledge still does matter in medical science and technology, as this study demonstrates.

Secondly, the authors have used those skills and existing knowledge to condense data, reducing the number of inputs to the machine algorithm to those that contain information, thereby increasing the probability that it produces something robust and useful, at the same time reducing the needed computational resources and the required number of cases to learn from. Although effective in general, this also has the risk of omissions or information destruction by non-expert handling.^6^ For instance, the use of an average beat omits arrhythmia information and destroys beat-to-beat variability; atrial depolarization information was absent in the input to the model. In theory, that could have influenced the results, in particular for the AF and VA outcome associations.

Thirdly, the data used in this study have been collected prospectively and specifically for research purposes, in contrast to many ML studies that have used existing, real-world, large archives from medical institutions. Those archives have been created for a variety of reasons, administrative, ease of information access, billing purposes, quality control, and others, but research use was mostly either an afterthought or a minor motivation. As a result, data are often ‘dirty’ and incomplete, institution-specific, and hard to generalize. The UK Biobank,^2^ with all its shortcomings and size restrictions, offers better prospects for data quality and generalizability.

Fourthly, the objective of the study is specific and well defined: to contribute to a physician’s decision on long-term monitoring and treatment for a very common condition, CAD. Certainly, having an additional risk indicator for the development of HF by itself is not a treatment-changing paradigm, but it may help in the decision-making process. In addition, the study suggests the possibility of using single-lead ECG devices, such as in smart watches and specific over-the-counter devices, now commonly used for self-guided arrhythmia monitoring, also for monitoring the progression of CAD. Clearly, this is just an initial study, and there are many possible roadblocks for such intended use (not in the least the signal processing and signal quality of the devices), but it points to an untapped potential of these devices in a well-defined higher-risk patient group.

Fifthly, the researchers, instead of using supervised ML which depends on outcomes, took the decision to use the method of clustering. The analysis of the clusters found can provide information that goes beyond the association of cluster membership and specific outcomes for which the authors have used it.^6^ For example, it gives a multidimensional (instead of single feature) definition of what is a ‘normal’ ECG morphology in the population considered. All ECG morphologies that belong to Cluster 2 in this study are to be considered normal, even though they may have widely varying metrics in any of the principal dimensions (e.g. width, amplitude, and form). ECGs in Cluster 1 are abnormal, in various ways; the authors did not define more than one distinct abnormal cluster, although they clearly could have done so with more data at their disposal, as can be induced from their figures 2 and supplementary 1. As such, the authors cannot conclude more than ‘individuals with abnormal morphology of ECG lead I (paraphrased by me) were at a higher prevalent and incident HF risk’, but with more data and more clusters at our disposal, no doubt more than one typical ‘abnormal’ pattern would emerge, with different clinical impacts. It is not surprising that the variability of feature values in the ‘abnormal’ cluster is higher than in the ‘normal’ cluster.

Lastly, this study concentrated on ECG lead I only, which likely does not give the whole picture. The inclusion of leads with other angles of visibility could have discovered more at-risk patients, likely with other affected arteries. This is certainly a limitation of the study, although not of the methodology. It would be interesting to understand how important the inclusion of other (orthogonal) leads is for the allocation of a patient to one or the other cluster.

Although the study by Madrid *et al*. is relatively simple and the results may not be spectacular or totally surprising, it is well conducted and clearly reported. It provides answers while also raising further questions, as all good science does, inspires further research and is well worth reading. There is no doubt that modern clustering techniques, expertly used, have the potential for better patient classification and can increase our understanding of disease phenotypes.

## Data Availability

No new data were generated or analysed in support of this research.

## Lead author biography

Johan de Bie obtained a PhD in Applied Physics from the Delft University of Technology, The Netherlands, in 1985, after which he became technical director of the Heart Station at Leiden University Hospital. He later moved to Italy and industry at a major manufacturer of ECG equipment, Mortara Instrument, where he led engineering and algorithm development teams until 2021. He continued to participate in scientific research through the University of Bologna and the Computing in Cardiology and International Society for Computerized Electrocardiology organizations. He now is part-time adjunct professor at the University of Bologna and consultant of medical device industries.
